# Serum miRNAs Predicting Sustained HBs Antigen Reduction 48 Weeks after Pegylated Interferon Therapy in HBe Antigen-Negative Patients

**DOI:** 10.3390/ijms19071940

**Published:** 2018-07-02

**Authors:** Koji Fujita, Shima Mimura, Hisakazu Iwama, Mai Nakahara, Kyoko Oura, Tomoko Tadokoro, Takako Nomura, Joji Tani, Hirohito Yoneyama, Asahiro Morishita, Makoto Oryu, Takashi Himoto, Hironori Nishitsuji, Kunitada Shimotohno, Masao Omata, Tsutomu Masaki

**Affiliations:** 1 Department of Gastroenterology and Neurology, Faculty of Medicine, Kagawa University, 1750-1 Ikenobe, Miki, Kita, Kagawa 761-0793, Japan; 92m7v9@med.kagawa-u.ac.jp (K.F.); shima@med.kagawa-u.ac.jp (S.M.); m-nakahara@med.kagawa-u.ac.jp (M.N.); kyoko_oura@med.kagawa-u.ac.jp (K.O.); t-nishioka@med.kagawa-u.ac.jp (T.T.); takako-n@med.kagawa-u.ac.jp (T.N.); j.tani@yashima-hp.com (J.T.); hyoneyam@med.kagawa-u.ac.jp (H.Y.); asahiro@med.kagawa-u.ac.jp (A.M.); oryum@saiseikai-kagawa.jp (M.O.); 2 Life Science Research Center, Kagawa University, 1750-1 Ikenobe, Miki, Kita, Kagawa 761-0793, Japan; iwama@med.kagawa-u.ac.jp; 3 Department of Medical Technology, Kagawa Prefectural University of Health Sciences, 281-1 Hara, Mure, Takamatsu, Kagawa 761-0123, Japan; imoto@chs.pref.kagawa.jp; 4 Research Center for Hepatitis and Immunology, National Center for Global Health and Medicine, 1-7-1 Kohnodai, Ichikawa, Chiba 272-8516, Japan; lbnishitsuji@hospk.ncgm.go.jp (H.N.); lbshimotohno@hospk.ncgm.go.jp (K.S.); 5 Department of Gastroenterology, Yamanashi Prefectural Central Hospital, 1-1-1 Fujimi, Kofu, Yamanashi 400-8506, Japan; aug8808@yahoo.co.jp

**Keywords:** hepatitis B surface antigens, hepatitis B virus, interferons, microRNAs, solute carrier proteins

## Abstract

The therapeutic goal for hepatitis B virus (HBV) infection is HBs antigen (HBsAg) seroclearance, which is achieved through 48-week pegylated interferon (Peg-IFN) therapy. This study aimed to identify predictive biomarkers for sustained HBsAg reduction by analyzing serum microRNAs. Twenty-two consecutive chronic HBV infection patients negative for HBe antigen (HBeAg) with HBV-DNA levels <5 log copies/mL, alanine aminotransferase (ALT) <100 U/L, and compensated liver functions, were enrolled. The patients were subcutaneously injected with Peg-IFNα-2a weekly for 48 weeks (treatment period), followed by the 48-week observation period. HBsAg 1-log drop relative to baseline levels recorded at the end of the observation period was considered effective. Sera were obtained at weeks 0 and 24 during the treatment period analyzed for microRNAs. The microRNA (miRNA) antiviral activity was evaluated in vitro using Huh7/sodium taurocholate cotransporting polypeptide (NTCP) cells. As a result, six patients achieved the HBsAg 1-log drop after the observation periods. Comparison of serum microRNA levels demonstrated that high miR-6126 levels at week 24 predicted HBsAg 1-log drop. Furthermore, miR-6126 reduced HBsAg in culture medium supernatants and intracellular HBV-DNA quantities in Huh7/NTCP cells. In conclusion, high serum miR-6126 levels during Peg-IFN therapy predicted the HBsAg 1-log drop 48 weeks after the completion of therapy. In vitro assays revealed that miR-6126 was able to suppress HBsAg production and HBV replication.

## 1. Introduction

The therapeutic goal for hepatitis B virus (HBV) infection is seroclearance of HBs antigen (HBsAg) [1]. HBsAg levels are correlated with hepatocellular carcinoma (HCC) occurrence in HBe antigen (HBeAg)-negative patients [2]. The spontaneous HBsAg seroclearance rate is estimated at 1.15% per year [3]. Although daily administration of nucleos(t)ide analogues (NUCs) reduces serum HBV-DNA levels and induces HBeAg seroconversion [4,5], reduction of serum HBsAg levels remains the emerging therapeutic target [6,7,8].

The evidence of HBsAg reduction through pegylated interferon (Peg-IFN) therapy is well established in HBeAg-positive [9,10,11] and HBeAg-negative patients [12,13,14,15,16,17] with baseline HBV-DNA levels >5 log copies/mL. Furthermore, a randomized, controlled, open-label trial demonstrated the efficacy of Peg-IFN therapy in decreasing HBsAg in a proportion of patients negative for HBeAg with sustained undetectable plasma HBV-DNA levels, though this study was not able to present a significant increase in HBsAg clearance rates in the Peg-IFN-added to the NUC group compared with the NUC alone group [18]. Nevertheless, Peg-IFN therapy takes 48 weeks or longer [19], and several adverse effects result from Peg-IFN injection in patients with or without NUCs. Thus, biomarkers predicting the efficacy of one-year therapeutic course, as well as sustained serological and virological responses, remain to be identified.

MicroRNAs (miRNAs) are small non-coding RNAs of 20–25 nucleotides in length [20]. miRNAs bind to messenger RNAs at their 3′-untranslated region and inhibit their translation, thereby regulating gene expression post-transcriptionally and modulating biological processes, such as intracellular metabolism, cell proliferation, differentiation, apoptosis, and angiogenesis [21,22,23]. Recently, circulating miRNAs have been investigated as serum biomarkers for diagnosis and prognosis in various diseases [24].

Here, we report serum miRNAs identified during Peg-IFN therapy that are able to predict sustained HBsAg reduction relative to baseline levels 48 weeks after the completion of therapy in HBeAg-negative chronic HBV infection patients with serum HBV-DNA levels <5 log copies/mL and compensated liver functions. Furthermore, we showed that one of the miRNA’s presented antiviral activity in vitro.

## 2. Results

### 2.1. Study Cohort at Baseline

Twenty-two consecutive patients met the inclusion criteria and were enrolled in the study (Table 1). The patient population consisted of thirteen males and nine females. The NUC group continued to take NUCs throughout the 48-week treatment and 48-week observation periods. The median HBsAg levels at baseline were 674.02 U/mL (minimum 80.13 U/mL and maximum 19,099.89 U/mL) for the NUC group and 73.80 U/mL (35.01–6179.34 U/mL) for the monotherapy group, with no statistically significant difference in HBsAg levels between the two groups (by Mann–Whitney *U* test). The median age, ALT, platelet count, and Fibrosis-4 (Fib-4) index at baseline were also not significantly different between the two groups.

HBV-DNA levels were <2.1 or <2.1+ log copies/mL in all of the twelve patients belonging to the NUC group. Among the patients who underwent IFN monotherapy, nine of the ten individuals (with the exception of case 6) presented baseline HBV-DNA levels >2.1 log copies/mL, with a median of 3.4 log copies/mL (2.2–4.6 log copies/mL). HBV genotyping was performed in twenty patients; genotype C was identified in eighteen patients, and genotype B was found in two patients. All patients were negative for HBeAg.

### 2.2. HBsAg Levels during the Treatment (Weeks 0–48) and Observation (Weeks 49–96) Periods

**Figure 1:**
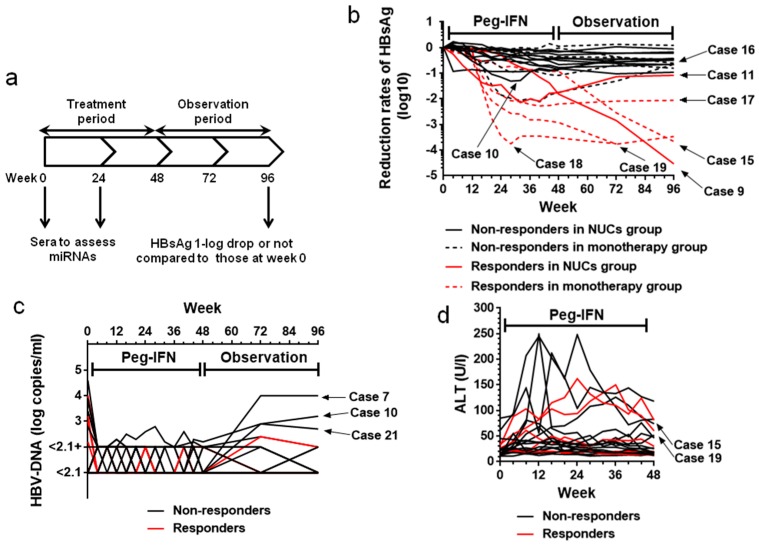
The clinical course of the patients through the treatment and observation periods. (**a**) Study design; (**b**) Reduction rates of serum HBs antigen (HBsAg) levels from baseline through the 48-week treatment and 48-week observation periods (black lines for non-responders in the nucleos(t)ide analogues (NUC) group; black dotted lines for non-responders in the monotherapy group; red lines for responders in the NUC group; red dotted lines for responders in the monotherapy group). Immediately after the completion of therapy, HBsAg 1-log drop was observed in seven of the twenty-two patients (cases 9, 10, 11, 16, 17, 18 and 19), while the other fifteen patients failed. Within the observation period following the treatment period, a single patient (case 15) achieved HBsAg 1-log drop. In contrast, two (cases 10 and 16) of the seven patients who presented HBsAg 1-log drop at the end of the treatment period experienced recovery in HBsAg levels and failed to maintain the reduction. Thus, the total number of responders was six (cases 9, 11, 15, 17, 18 and 19). Among these patients, cases 9 and 11 belonged to the NUC group (red lines), and the other four patients were in the monotherapy group (red dotted lines); (**c**) Hepatitis B virus (HBV)-DNA levels throughout the treatment and observation periods (black lines for non-responders and red lines for responders). All of the twelve patients in the NUC group went through the treatment and observation periods showing HBV-DNA levels <2.1 or <2.1+ log copies/mL. In the monotherapy group, nine of the ten patients completed the treatment period with HBV-DNA levels <2.1 or <2.1+ log copies/mL. Case 10, whose baseline HBV-DNA level was 3.9 log copies/mL, experienced a viral response, demonstrated by a reduction of the serum HBV-DNA level to <2.1 at week 40, but showed recovery up to 2.2 log copies/mL by week 48. At the end of the study, seven of the ten patients exhibited HBV-DNA <2.1 or <2.1+ log copies/mL. Three patients (cases 7, 10 and 21) finished the study with HBV-DNA >2.1 log copies/mL, but their HBV-DNA levels at the end of the study did not surpass their baseline levels; (**d**) Alanine aminotransferase (ALT) levels increased to >100 U/mL in six patients, but did not surpass 250 IU/mL throughout the treatment period. Six responders are highlighted with red lines, including cases 15 and 19, which experienced transient ALT increases >100 U/mL. Black lines represent non-responders. miRNA, microRNA; Peg-IFN, pegylated interferon.

The clinical course of patients’ HBsAg levels is shown in Figure 1b (black lines for non-responders in the NUC group; black dotted lines for non-responders in the monotherapy group; red lines for responders in the NUC group; red dotted lines for responders in the monotherapy group). Immediately after the completion of therapy, HBsAg 1-log drop was observed in seven of the twenty-two patients—cases 9, 10, 11, 16, 17, 18 and 19 (Table 1)—whereas the other fifteen patients failed.

Within the observation period following the treatment period, a single patient (case 15) achieved HBsAg 1-log drop. In contrast, two (cases 10 and 16) of the seven patients who presented HBsAg 1-log drop at the end of the treatment period experienced recovery of HBsAg levels and failed to maintain the reduction. Thus, the total number of responders was six (cases 9, 11, 15, 17, 18 and 19) (Figure 1b). Among these patients, cases 9 and 11 belonged to the NUC group (red lines), while the other four patients were in the monotherapy group (red dotted lines). The median HBsAg levels at baseline were 238.34 U/mL (45.86–851.20 U/mL) for responders and 710.38 U/mL (35.01–19,099.89 U/mL) for non-responders, with no statistically significant difference between the two groups (by Mann–Whitney *U* test). The median age, ALT, and platelet counts at baseline were also not significantly different between the two groups. The median Fib-4 index was significantly higher in responders than in non-responders at 2.049 for responders and 1.279 for non-responders (*p* < 0.05 by Mann–Whitney *U* test). NUC intake did not significantly correlate with HBsAg 1-log drop at week 96 (by Fisher’s exact test).

Initial doses of Peg-IFN were 180 μg for nine patients. Among them, three patients (cases 2, 6 and 7) completed the treatment course without dose reduction or adverse events more severe than grade 1, but failed to achieve HBsAg 1-log drop. Peg-IFN doses were reduced to 90 μg for the other six patients because of grade 2 to 3 white blood cell decreases or grade 2 anemia. Thirteen patients completed the treatment period with Peg-IFN with 90 μg without dose reduction.

### 2.3. HBV-DNA Levels During Treatment (Weeks 0–48) and Observation (Weeks 49–96) Periods

The clinical course of the patients’ HBV-DNA levels is shown in Figure 1c (black lines for non-responders and red lines for responders). All of the twelve patients in the NUC group went through the treatment and observation periods showing HBV-DNA levels <2.1 of <2.1+ log copies/mL.

In the monotherapy group, nine of the ten patients completed the treatment period with HBV-DNA levels <2.1 or <2.1+ log copies/mL. Case 10, whose baseline HBV-DNA level was 3.9 log copies/mL, experienced a viral response, demonstrated by a reduction of the serum HBV-DNA level <2.1 log copies/mL at week 40, but showed recovery up to 2.2 log copies/mL by week 48. At the end of the study, seven of the ten patients exhibited HBV-DNA <2.1 or <2.1+ log copies/mL. Three patients, cases 7, 10, and 21, finished the study with HBV-DNA >2.1 log copies/mL, but their HBV-DNA levels at the end of the study did not surpass their baseline levels.

### 2.4. Transient Increase in ALT Levels

The clinical course of ALT levels during the treatment period is shown in Figure 1d (black lines for non-responders and red lines for responders). Increases in ALT levels >100 U/L were observed in six patients (cases 4, 10, 15, 16, 19 and 21), which did not surpass 250 U/mL. Among these patients, cases 15 and 19 belonged to responders, while the other four patients were non-responders. Episodes of ALT increases >100 U/L did not significantly correlate with HBsAg 1-log drop (by Fisher’s exact test).

### 2.5. miRNA Expression Analysis

**Figure 2:**
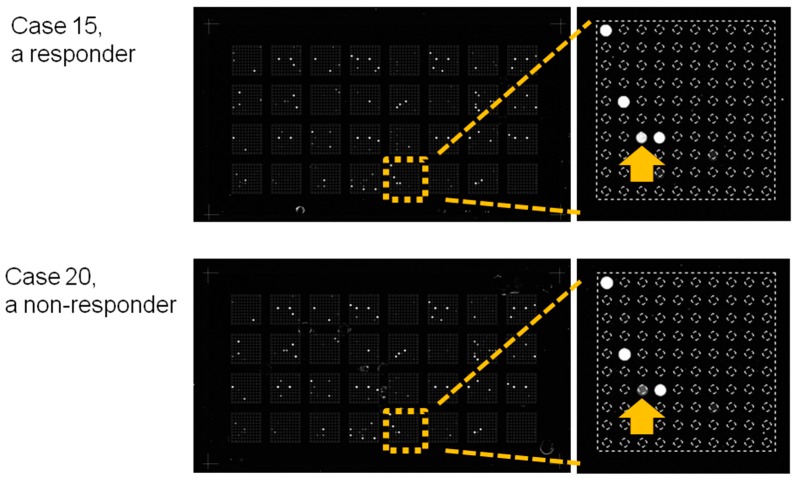
Images of the microarray chips. Sera obtained at week 24 were loaded onto the miRNA microarray chips. Yellow squares indicate blocks in which DNA-probes specific to miR-6126 were added to the microarray chips. The signal corresponding to miR-6126 is depicted by yellow arrows for case 15, a responder; and case 20, a non-responder. The signal intensity of case 15 was stronger, while that of case 20 was weaker.

To investigate candidate miRNAs that may serve as predictive biomarkers for Peg-IFN-therapeutic responses, we screened miRNA expression levels in serum samples collected at weeks 0 and 24 during the treatment period by microarray analyses. The comparison of miRNA expression profiles between responders and non-responders at week 24 using an FDR < 10% revealed 4 of 2565 miRNAs (miR-4878-3p, miR-6126, miR-6769a-3p, and miR-1203) that were differentially expressed between the two groups (Table 2). Images of the microarray chips for representative patients are shown in Figure 2. Yellow squares indicate blocks in which DNA-probes specific to miR-6126 were added to the microarray chips. The signal corresponding to miR-6126 is indicated with yellow arrows for case 15, a responder; and case 20, a non-responder. The signal intensity in case 15 was stronger, while that in case 20 was weaker. No miRNAs identified in sera from week 0 presented significantly different signal intensities between the two groups (by false discovery rate (FDR) < 10%).

**Figure 3:**
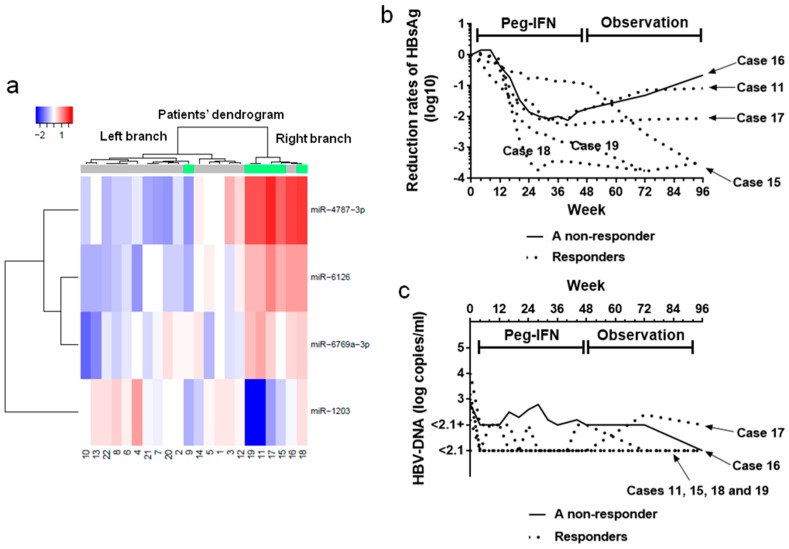
miRNA microarray analysis. (**a**) Hierarchical clustering of miRNA expression profiles in serum samples at week 24. Samples are arranged in columns, and miRNAs are arranged in rows. The dendrogram on the left of the heatmap represents miRNA clustering. The dendrogram above the heatmap represents sample clustering. The right branch of the dendrogram above the heatmap includes five of the six responders and a non-responder. Sample identity (ID) numbers are indicated below the heatmap. The heatmap represents the relative signal intensity of each miRNA, in which the log_2_ intensity is median-centered for each row. The color-coding is indicated with a horizontal bar on the bottom left; (**b**) Reduction rates in serum HBsAg levels from baseline during the treatment and observation periods. The five responders (dotted lines) and the non-responder (a line) in the right branch of the dendrogram above the heatmap are included in this figure. HBsAg level of the non-responder is represented by a line, and those of the five responders are represented by dotted lines. The non-responder, case 16, had achieved HBsAg 1-log drop once by the end of the treatment but failed to maintain this decrease throughout the observation period; (**c**) HBV-DNA levels of the five responders and the non-responder are included in the right branch of the dendrogram above the heatmap, throughout the treatment and observation periods. The serum HBV-DNA levels in the non-responders is represented by a line, and those in five responders are represented by dotted lines. The non-responder, case 16, experienced a reduction in serum HBV-DNA level below the detection threshold by the end of observation period, although the HBsAg level increased during the observation period.

Cluster analyses demonstrated that the circulating miRNAs detected at week 24 clustered differentially between responders and non-responders (Figure 3a), which is represented by a dendrogram above the heatmap. The right branch of the dendrogram included five of the six responders and one non-responder (case 16). Case 16 had achieved HBsAg 1-log drop once by the end of the treatment but failed to maintain this decrease during the observation period (Figure 3b, a line), even though this patient experienced a decrease in serum HBV-DNA levels below the detection limit at week 96 (Figure 3c, a line). The five responders are illustrated by dotted lines in Figure 3b,c.

**Figure 4:**
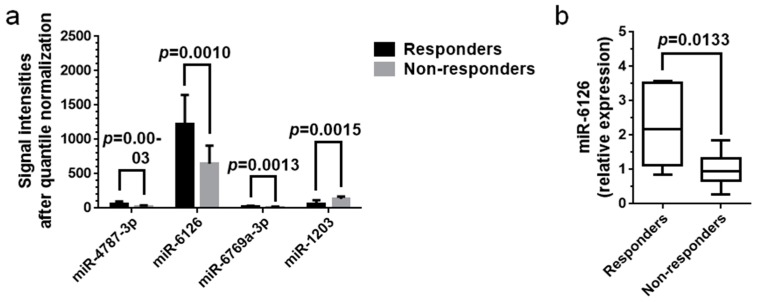
Validation of serum miRNA levels via real-time qPCR. (**a**) Average signal intensities of miRNAs in week-24 sera after quantile normalization of raw data. Although each miRNA was significantly differentially expressed between responders and non-responders (*p* < 0.05), the signal intensities for miR-6126 were the strongest, and those for the other miRNAs were much weaker. The data suggested that miR-6126 was the most abundant miRNA in patient sera among the four miRNAs. The data were analyzed with Student’s *t*-tests and plotted with the mean and SD; (**b**) Real-time qPCR analysis validated the significantly higher expression of serum miR-6126 in the six responders compared with that in the sixteen non-responders (*p* < 0.05). The other three miRNAs were not detected via real-time qPCR. Levels of serum miRNAs were normalized to the levels of cel-miR-39, an external control. Data were analyzed with Mann–Whitney *U* test and are presented as the median and range.

The signal intensities of the four miRNAs (miR-4878-3p, miR-6126, miR-6769a-3p, and miR-1203) after quantile normalization of their raw data are presented in Figure 4a. Although the signal intensities were significantly different between responders and non-responders for each miRNA (*p* < 0.05), the normalized values for miR-6126 were much higher than those for other miRNAs. The data suggested that miR-6126 was the most abundant miRNA in the patient sera relative to the other three miRNAs. The relative expression levels of miR-6126 were validated using real-time qPCR. The median level of miR-6126 in responders was significantly higher than in non-responders, suggesting predictive potential of this miRNA for a sustained HBsAg reduction after Peg-IFN therapy (*p* < 0.05, Figure 4b). The median CT value for miR-6126 in the twenty-two patients was 27.13 (25.00–31.51), while that for cel-miR-39 was 24.23 (23.35–26.64), indicating that miR-6126 in the serum samples was diluted approximately 2^2.9^ times compared with cel-miR-39. The median CT values for cel-miR-39 were not significantly different between responders (24.26 (23.87–24.66)) and non-responders (24.23 (23.35–26.64)) by Mann–Whitney *U* test. Real-time qPCR was also performed for miR-6769a-3p, miR-4787-3p, and miR-1203, but failed to reveal changes in cDNA levels because the signal intensities of the three miRNAs were below the detection threshold.

### 2.6. Virological Effects of miR-6126 against HBV

The virological effects of miR-6126 against HBV were determined using Huh7/sodium taurocholate cotransporting polypeptide (NTCP) cells infected with genotype C wild-type HBV. HBV infection of Huh7/NTCP cells was validated based on intracellular HBV-DNA quantities, HBV-DNA levels determined in culture medium supernatants via real-time qPCR and HBsAg levels detected in culture medium supernatants by ELISA. The HBV-infection group presented significantly larger HBV-DNA quantities inside the cells (Figure S1a) and higher HBV-DNA (Figure S1b) and HBsAg (Figure S1c) levels in culture medium supernatants than the mock group (*p* < 0.05). Increased intracellular miR-6126 quantities observed after transfection of the miR-6126 mimic into Huh7/NTCP cells was also determined via real-time qPCR. The intracellular miR-6126 quantities were significantly larger in the miR-6126 mimic-transfected group than in the negative control (NC) mimic-transfected group (*p* < 0.05, Figure S1d).

The complete HBV genome was searched for sequences complementary to miRNAs using miRBase to speculate whether miR-6126 binds to any regions in HBV-DNA directly. As a result, no complementary regions were detected to human mature miR-6126 or human pri-mir-6126 in the HBV complete genome.

**Figure 5:**
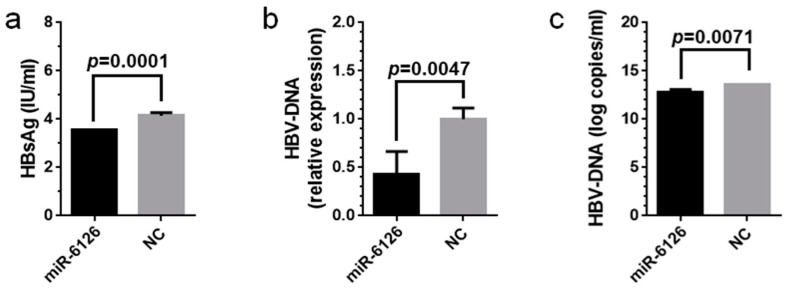
Antiviral activity of miR-6126 in vitro. (**a**) Transfection of miR-6126 mimic into Huh7/sodium taurocholate cotransporting polypeptide (NTCP) cells significantly reduced HBsAg levels in culture medium supernatants (*p* < 0.05); (**b**,**c**) Transfection of miR-6126 mimic into Huh7/NTCP cells significantly reduced intracellular HBV-DNA quantities (**b**) and HBV-DNA levels in culture medium supernatants (**c**) compared with the negative control (NC) mimic (*p* < 0.05). In the experiment (**a**), cells were passaged on day 0; miR-6126 mimic or NC mimic was transfected on day 1; cells were infected with HBV on day 2; and culture medium supernatants were harvested on day 5. In the experiments (**b**,**c**), cells were passaged on day 0; miR-6126 mimic or NC mimic was transfected into cells on day 1; cells were infected with HBV on day 2; and miR-6126 mimic or NC mimic was added to cells again on days 3, 5 and 7. Intracellular DNA and culture medium supernatants were harvested on day 9. Intracellular HBV-DNA quantities were normalized to those of GAPDH, an internal control. All the experiments (**a**–**c**) were performed three times with four replicates and analyzed with Student’s *t*-tests, then the representative data were plotted with the mean and SD.

Transfection of the miR-6126 mimic into cells resulted in significantly lower HBsAg levels in the culture medium supernatants than transfection of the NC mimic (*p* < 0.05, Figure 5a). In this experiment, Huh7/NTCP cells were passaged on day 0; the miR-6126 mimic or NC mimic was transfected into cells on day 1; cells were infected with HBV on day 2; and culture medium supernatants were harvested on day 5.

Intracellular HBV-DNA quantities (Figure 5b) and HBV-DNA levels in the culture medium supernatants (Figure 5c) were also significantly lower in the miR-6126 mimic-transfected group than in the NC mimic-transfected group (*p* < 0.05). In this experiment, Huh7/NTCP cells were passaged on day 0; miR-6126 mimic or NC mimic was transfected into cells on day 1; cells were infected with HBV on day 2; and miR-6126 mimic or NC mimic was added to cells again on days 3, 5 and 7. Cellular DNA and culture medium supernatants were harvested on day 9.

## 3. Discussion

In the current study, Peg-IFN therapy induced HBsAg 1-log drop by the end of the treatment in seven of the twenty-two patients negative for HBeAg with HBV-DNA levels <5 log copies/mL. After the 48-week observation period, HBsAg 1-log drop was observed in six patients. Among the six patients, five patients experienced sustained serological responses; that is, HBsAg 1-log-drop was maintained over the one-year observation period. Serum HBV-DNA levels were maintained at <2.1 or <2.1+ log copies/mL in the NUC group until completion of the observation period. In the monotherapy group, seven of the ten patients completed the study with HBV-DNA levels <2.1 or <2.1+ log copies/mL. The comparison of serum miRNA levels between the six responders and sixteen non-responders at week 24 via microarray analyses revealed four miRNAs that were significantly differentially expressed between the two groups. Real-time qPCR analyses demonstrated that miR-6126 levels were higher in the sera of responders than in those of non-responders. Furthermore, miR-6126 exhibited virological effects by reducing HBsAg and HBV-DNA levels in culture medium supernatants and decreasing HBV-DNA quantities in Huh7/NTCP cells.

Two patients, cases 10 and 16, experienced HBsAg 1-log drop by the end of the treatment period but failed to maintain the decrease through the observation period (Figure 1b). Among these two patients, case 16 showed relatively high expression of miR-6126, similar to that in the five responders, and clustered with the responders (Figure 3a). Case 10 experienced recovery of both HBsAg and HBV-DNA levels, ending in failure to achieve HBsAg 1-log drop and a reduction in HBV-DNA levels <2.1 log copies/mL (Figure 1b,c). Therefore, although miR-6126 partially works by suppressing HBV replication, other factors may also be necessary for the antiviral effects of Peg-IFN.

Case 9, a responder, exhibited a low miR-6126 signal similar to those of non-responders (Figure 3a). Case 9 was unique among responders because this patient experienced a viral breakthrough and was prescribed adefovir (ADV) with lamivudine (LMD) before being enrolled in the study (Table 1). Recently, nucleoside analogues have been reported to differ from nucleotide analogues in their mechanism of antiviral activity via the induction of IFN-λ3 expression [25]. However, whether the phosphorylation of nucleosides affects the antiviral mechanisms of Peg-IFN in combination with NUCs is a question that is open to further investigation.

Comparing the baseline characteristics of responders and those of non-responders, the Fib-4 index of responders was higher than that of non-responders. Liver fibrosis is recognized as a limiting factor for Peg-IFN therapy because cirrhotic patients are likely to present adverse effects of the treatment [7]. However, the predictive value of liver fibrosis staging for the efficacy of Peg-IFN therapy was not significant in a past study completing a 96-week Peg-IFN administration in HBeAg-negative patients [26]. Peg-IFN therapy for 48 weeks was at least shown to be effective in patients with more progressed liver fibrosis, as well as those with less progressed fibrosis, according to our data.

The changes in serum/plasma miRNAs in the presence of HBV infection have been well studied. In comparison with miRNAs in the serum/plasma of healthy controls, liver-specific miRNAs, including miR-122 family members, miR-99a-5p, and miR-192-5p, are highly expressed in the serum/plasma of HBV-infected patients [27,28]. In a past study, liver-specific miRNAs in the serum were found to significantly decrease after the completion of 24-week Peg-IFN therapy relative to corresponding baseline levels [29]. The current study identified potential serum miRNAs during 48-week Peg-IFN therapy that could predict off-treatment efficacy. One of these miRNAs, miR-6126, is not specific to the liver. The reason that our results included miR-6126, while past reports did not, might simply be that, unlike the other studies, we analyzed 2565 miRNAs that included miR-6126. Although serum miR-6126 might play pivotal roles in decreasing and clearing HBsAg, information regarding miR-6126 is limited at present. Thus, the cell types expressing high levels of miR-6126 and the role of miR-6126 in the replication of HBV or immune responses specific to HBV infection remain to be elucidated. A recent study demonstrated that miR-6126 acts as a tumor-suppressive miRNA in ovarian cancer cells [30], but no reports have mentioned the role of miR-6126 in suppressing HBV replication.

With regard to longitudinal analysis of serum miRNAs, miRNAs that presented significant expression changes between week 0 and 24 during Peg-IFN therapy were also assessed. However, no miRNAs were extracted in that analysis, partially because miRNA expression profiles at baseline might vary patient by patient. Assuming that miRNA profiling at baseline present too much variability patient by patient, a longitudinal analysis comparing before and after treatment period might not identify miRNAs with significant expression changes between the two time points. Once you initiate Peg-IFN therapy, baseline variability of miRNAs should be canceled and the difference of miRNA profiles induced by Peg-IFN should be highlighted according to the therapeutic responses of patients.

In the current study, the antiviral effects of miR-6126 were validated in the human NTCP-1-overexpressing Huh7 cell line. Human NTCP-1 has been identified as a major HBV receptor on the surface of hepatocytes [31]. NTCP-1-overexpressing hepatoma cell lines have been widely employed for basic research; two of these cell lines in particular, HepG2 and Huh7, are generally used in experiments. Although HepG2/NTCP cells have been reported to be superior to Huh7/NTCP cells in terms of HBV infectivity [32], in the current study, Huh7/NTCP cells were selected because the combination of Huh7 cells and genotype C wild-type HBV more closely mimics Japanese patients infected with HBV than do HepG2 cells. Huh7 is a well-differentiated hepatoma cell line derived from a Japanese patient [33], while HepG2 is a hepatoblastoma cell line derived from a Caucasian patient [34]. In addition, genotype C is the predominant form of HBV in Japan, although the emergence of genotype A has been increasing in acute hepatitis patients [35,36]. In this study, HBV infection was established in Huh7/NTCP cells generated by Nishitsuji et al. [37] via the administration of five genome equivalents of virus per cell (Figure S1a).

Notably, the anti-HBV replication activity of miR-6126 observed in vitro must be carefully translated to a clinical setting, as the antiviral activity of miR-6126 was evaluated using miR-6126 mimic at 50 nM concentration in transfection assays. However, miR-6126 concentration in serum was found to be more diluted than that of cel-miR-39 (approximately 29.2 pM in a 200-µL-serum sample). This discrepancy might be partially caused by the difference between clinical samples and in vitro assessment.

Some of stopping rules in Peg-IFN therapy are preferable to support patient’s benefit and to save costs. J. Vlachogiannakos and G.V. Papatheodoridis proposed on-treatment predictions of negative viral responses, a lack of decrease of HBV-DNA >2 log copies/mL at week 12 [38]. HBsAg decrease >0.5 log IU/mL at week 12 was also proposed as a positive response prediction biomarker to achieve undetectable HBV-DNA levels [13]. However, we did not adopt stopping rules concerning negative or positive response prediction biomarkers by the two reasons as follows: (a) Peg-IFN therapy is reported that the therapy might have potential to suppress HCC occurrence in HBV-infected patients [39]; (b) Recently, two randomized controlled trials (RCTs) using Peg-IFN with nucleos(t)ide analogues to achieve HBsAg seroclearance were reported [17,18]. It seems that they did not adopt stopping rules for Peg-IFN therapy in perspectives of HBsAg decline or HBV-DNA decline at week 12, except for the dose reduction and discontinuation of Peg-IFN treatment to relieve adverse effects were described in the two studies.

To mention the definition of treatment response, one of the strictest definitions should be sustained HBsAg seroclearance 96 weeks after Peg-IFN therapy was finished [18]. However, seroclearance rates of HBsAg usually result in less than 10%. However, even a 10% decrease of HBsAg is worth being considered in patients with HBV-DNA <4 log copies/mL because the prevalence of HCC increases with positive correlation to serum HBsAg levels in patients with HBV-DNA <4 log copies/mL [2].

The first limitation of this study is its small sample size. A validation study using a larger cohort should be performed before applying the current data to the general population. Second, there was substantial heterogeneity in the patients’ background, as some patients underwent Peg-IFN therapy in combination with NUCs, whereas other patients were treated with Peg-IFN alone. Third, the cell types and organs that secrete miR-6126 in Peg-IFN-treated patients remain unknown. Fourth, whether miR-6126 in patient sera exhibits has anti-HBV activity needs to be determined, and its target molecules in HBV replication need to be clarified.

In conclusion, among 2565 miRNAs examined at week 24 during the 48-week Peg-IFN therapy, serum miR-6126 was identified as a candidate serum biomarker for predicting HBsAg 1-log drop 48 weeks after the completion of therapy. Furthermore, miR-6126 exhibited the potential to suppress intracellular HBV quantities in vitro. The present findings will support decision making in the selection of patients suitable to receive Peg-IFN therapy with the aim of HBsAg reduction.

## 4. Materials and Methods

### 4.1. Ethics Statement

This study was conducted in accordance with the ethical principles of the Declaration of Helsinki and was approved by the Institutional Review Board of Kagawa University Faculty of Medicine on 18 April 2013 (approval number, Heisei 22-063). Before this study was initiated, written informed consent was obtained from each patient to allow analyses of serum miRNAs.

### 4.2. Patients

Twenty-two consecutive adult patients with HBeAg-negative chronic HBV infection were enrolled in the study. Twelve of the patients were prescribed NUCs (NUC group), while the other ten patients were not (monotherapy group). Additional inclusion criteria were serum HBV-DNA levels <5 log copies/mL, alanine aminotransferase (ALT) levels <100 U/L, and compensated liver functions. The exclusion criteria were evident cirrhosis, psychiatric diseases, an absolute neutrophil count <1.0 × 10^9^/L, a platelet count <5.0 × 10^10^/L, and a history of alcohol or drug abuse. Fibrosis-4 index (Fib-4 index) was calculated using the following equation: age × AST (U/L)/(platelet count (10^9^/L) × √ALT (U/L)) [40].

### 4.3. Study Design

The patients were assigned to a single study arm and subcutaneously injected with Peg-IFNα-2a 90 or 180 μg weekly for 48 weeks (treatment period, weeks 0 to 48) (Figure 1a). Patients were followed for 48 weeks after the completion of therapy (observation period, weeks 49 to 96). Serum HBsAg and HBV-DNA levels were monitored every 12 weeks. Serum samples were obtained and preserved at weeks 0 and 24 during the treatment period for miRNA analyses.

Initial doses and dose reduction of Peg-IFN was left to the physician’s decision. However, physicians were encouraged to avoid grade 3 or greater severe adverse events throughout the treatment course.

### 4.4. Efficacy of Treatment

The primary endpoint was defined as HBsAg 1-log drop relative to baseline levels. The secondary endpoint was defined as decreases in serum HBV-DNA levels to <2.1 or <2.1+ log copies/mL. Responders to Peg-IFN therapy were defined as patients who experienced HBsAg 1-log drop at week 96, while the others were considered non-responders. HBsAg levels were determined using ARCHITECT^TM^ i2000 (Abbott Laboratories, Chicago, IL, USA). HBV-DNA levels were measured using Cobas^®^ TaqMan^®^ 48 Analyzer (Roche Molecular Systems, Branchburg, NJ, USA).

### 4.5. miRNA Microarray Analysis

Serum samples obtained at weeks 0 and 24 were analyzed to identify miRNAs that were able to predict HBsAg 1-log drop at week 96. Serum RNA was extracted using a Serum/Plasma miRNeasy Mini Kit (Qiagen, Venlo, The Netherlands) according to the manufacturer’s protocol. The RNA sample quality was evaluated with an Agilent 2100 Bioanalyzer (Agilent Technologies, Santa Clara, CA, USA) and typically showed A260/280 ratios between 1.9 and 2.1. The samples were labeled using miRCURY Hy3^TM^ Power Labeling Kit (Exiqon, Vedbaek, Denmark) and hybridized onto 3D-Gene^®^ Human miRNA Oligo Chip, version 21 (Toray, Tokyo, Japan), in which serum miRNAs were hybridized to oligo DNA probes, and then the 3′-ends of miRNAs were stained by fluorescent dyes. Scanning was performed with a 3D-Gene^®^ Scanner 3000 (Toray) using 3D-Gene^®^ Extraction software, version 1.2 (Toray), to read the raw image intensity. The raw data were analyzed using GeneSpring GX, version 10.0 (Agilent Technologies), and subjected to quantile normalization [41]. The relative expression of miRNAs was calculated between responders and non-responders. Hierarchical clustering was performed via the farthest neighbor method using Pearson’s product-moment correlation coefficients as the metric. The relative signal intensity of each miRNA was shown on a heatmap, in which the log_2_ intensity was median-centered for each row. The microarray data were deposited in the NCBI Gene Expression Omnibus (GEO) under accession number GSE97506.

### 4.6. Real-Time qPCR for Serum miRNAs

Reverse transcription and real-time quantitative PCR (qPCR) were performed for serum miRNAs using the ΔΔCT method to confirm the differences in their expression observed through microarray analyses. TaqMan^®^ miRNA Assays (Thermo Fisher Scientific, Waltman, MA, USA) were used to determine the expression levels of miRNAs employing cel-miR-39 as an exogenous control (Assay ID: 475618 for miR-6126 and 000200 for cel-miR-39) [42]. Briefly, 200 μL serum was mixed with cel-miR-39 (3.5 × 10^8^ copies). Total RNA was extracted from the serum samples using the miRNeasy Serum/Plasma Kit and diluted to 1.0 ng/μL. Reverse transcription was performed in a 15-μL reaction mixture consisting of RNA 5 μL, 5× RT primer 3 μL and reverse transcription master mix 7 μL using the TaqMan^®^ miRNA Reverse Transcription Kit (Thermo Fisher Scientific). qPCR was performed in a final volume of 20 μL containing cDNA 2 μL, 20× qPCR mixture 1 μL, nuclease-free water 7 μL, and TaqMan^®^ Fast Advanced Master Mix (Thermo Fisher Scientific) 10 μL, according to the manufacturer’s protocol. cDNA was amplified and quantified using StepOnePlus^TM^ (Thermo Fisher Scientific).

### 4.7. Cell Line and Culture

Huh7/NTCP cells, a sodium taurocholate cotransporting polypeptide (NTCP)-1-overexpressing hepatoma cell line, were a gift of Kunitada Shimotohno (Research Center for Hepatitis and Immunology, National Center for Global Health and Medicine. Ichikawa, Japan). Huh7/NTCP cells are a Huh7-derived cell line transduced by pCAN-NTCP-myc and susceptible to HBV infection [37]. Cells were cultured in DMEM (Thermo Fisher Scientific) supplemented with 10% fetal bovine serum, 100 U/mL penicillin, 100 μg/mL streptomycin, and 250 μg/mL G418.

### 4.8. HBV Infection

Genotype C wild-type HBV (clone: C_JPNAT) was purchased from Phoenix Bio (Hiroshima, Japan). Huh7/NTCP cells were seeded at a density of 2.5 × 10^5^ cells/well in 0.5 mL/well culture medium in a 24-well plate. The cells were infected with five genome equivalents of virus/cell in the presence of 4% PEG8000 and 2% DMSO overnight [37].

### 4.9. HBV Replication Analysis

To determine intracellular HBV-DNA quantities, total DNA was extracted from HBV-infected Huh7/NTCP cells using PureLink^®^ Genomic DNA Mini Kit (Thermo Fisher Scientific) according to the manufacturer’s protocol. Total DNA was diluted to 10 ng/μL. Real-time qPCR was carried out using Fast SYBR^®^ Green Master Mix (Thermo Fisher Scientific), and fluorescent signals were analyzed using StepOnePlus^TM^. The primers used in the reactions were as follows: 5′-CCTCTGCCTAATCATCTCATGTTC-3′ (forward) and 5′-CGGTGTCGAGGAGATCTCGAATAG-3′ (reverse) for HBV; and 5′-CCATGCCATCACTGCCACCC-3′ (forward) and 5′-GCCAGTGAGCTTCCCGTTCAG-3′ (reverse) for GAPDH, as an internal control [37]. Each 20 μL reaction mixture consisted of 10 ng/μL DNA (2 μL), 50 μM each forward and reverse primers (0.12 μL), RNase-free water (7.76 μL), and Fast SYBR^®^ Green Master Mix 10 μL. The final concentration of each primer was 300 μM.

HBV-DNA levels in culture medium supernatants were determined using Cobas^®^ TaqMan^®^ 48 Analyzer. For sample preparation, a culture medium supernatant of 0.35 mL/sample was diluted with 1.55 mL PBS to obtain a total volume of 1.9 mL.

HBsAg levels in the culture medium supernatants were measured using the ARCHITECT^TM^ i2000.

### 4.10. Transfection of Cells with miRNA Mimics

miRNA mimics (Thermo Fisher Scientific) were transfected using Lipofectamine RNAiMAX^®^ Reagent (Thermo Fisher Scientific) and Opti-MEM^®^ (Thermo Fisher Scientific) according to the manufacturer’s protocol. The miR-6126 mimic (Assay ID: MC25200) and negative control (NC) mimic were used at a final concentration 50 nM to investigate the virological effects of miR-6126 against HBV replication and HBsAg production.

Intracellular quantities of miR-6126 were validated through reverse transcription and real-time qPCR using the ΔΔCT method. Total RNA was extracted from Huh7/NTCP cells using miRNeasy Mini Kit (Qiagen). The total RNA was then diluted to 1.0 ng/μL, and the miRNAs were reverse transcribed using the TaqMan^®^ miRNA Reverse Transcription Kit, according to the manufacturer’s protocol. The TaqMan^®^ miRNA Assays were performed to determine the expression levels of miR-6126 employing U6 (Assay ID: 001093) as an internal control. cDNA was amplified and quantified using StepOnePlus^TM^.

### 4.11. Statistical Analysis

Clinical data were either analyzed with Mann–Whitney U test and presented as the median and range or were analyzed with Fisher’s exact test. After quantile normalization, the miRNA microarray data were analyzed using Student’s *t*-tests and a false discovery rate (FDR) <10% and are presented as the mean and SD. Sequences of the HBV clone C_JPNAT were obtained from GeneBank (accession: AB246345, version: AB246345.1). HBV complete genome was searched for sequences complementary to the miRNAs using miRBase (http://www.mirbase.org/search.shtmL). In vitro experiments were performed three times with four replicates and then analyzed using Student’s *t*-tests, and representative data were plotted with the mean and SD. *p*-values < 0.05 were considered significant. The analyses were performed using GraphPad Prism 6 (GraphPad Software, La Jolla, CA, USA).

## Figures and Tables

**Table 1:** Baseline patient characteristics.

ID	Age	Sex	Body Weight (kg)	Initial Peg-IFN Dose (μg)	*NUCs	HBsAg (U/mL)	HBV-DNA (Log Copies/mL)	Genotype	ALT (U/mL)	Plate-Let (×10^9^/L)	Fib-4 Index
1	69	M	65	90	ETV	496.83	<2.1	C	23	119	2.418
2	45	M	68	180	ETV	341.94	<2.1	C	18	268	0.752
3	57	M	73	180	ETV	475.71	<2.1	C	13	190	1.581
4	42	M	71	90	ETV	132.36	<2.1	C	62	107	1.844
5	56	F	58	180	ETV	1255.75	<2.1	C	12	193	1.423
6	56	M	77	180	None	35.01	<2.1	†N.T.	45	239	1.048
7	43	M	66	180	None	6179.34	4.6	C	25	240	1.003
8	59	M	73	180	ETV	80.13	<2.1	C	16	208	1.135
9	49	M	63	90	LMD + ADV	851.20	<2.1	†N.T.	27	129	1.828
10	34	M	66	180	None	52.57	3.9	B	11	183	0.780
11	68	F	46	90	ETV	417.89	<2.1	C	16	84	5.262
12	54	F	63	90	ETV	2261.67	<2.1	C	11	158	1.855
13	52	M	70	180	ETV	923.92	<2.1	C	22	166	1.469
14	64	M	56	90	ETV	4528.94	<2.1+	B	23	163	1.965
15	45	F	83	180	None	45.86	2.2	C	32	127	2.722
16	41	F	63	90	None	4786.11	2.8	C	85	164	0.957
17	63	M	55	90	None	742.99	4.0	C	14	200	1.852
18	51	F	58	90	None	56.51	3.2	C	11	221	0.905
19	55	M	64	90	None	58.79	3.0	C	34	198	1.286
20	41	F	65	90	ETV	19099.89	<2.1	C	15	243	0.828
21	66	F	44	90	None	88.81	3.4	C	15	179	1.809
22	63	F	44	90	None	5470.72	3.8	C	14	150	2.245

*NUCs, nucleos(t)ide analogues; ETV, entecavir; LMD, lamivudine; ADV, adefovir; ID, identity; Peg-IFN, pegylated interferon; ALT, alanine aminotransferase; HBsAg, HBs antigen; Fib-4, fibrosis-4; M, male; F, female. †N.T., not tested.

**Table 2:** Serum miRNAs at week 24 predicting HBsAg 1-log drop after the observation period relative to HBsAg levels at baseline.

Name	Fold-Change	SD	*p*	Chromosomal Location
Up-regulated				
miR-4787-3p	2.881	1.270	0.0003	19
miR-6126	1.884	0.657	0.0010	16
miR-6769a-3p	1.602	0.378	0.0013	16
Down-regulated				
miR-1203	0.500	0.356	0.0015	17

## Supplementary Materials

Supplementary materials can be found at http://www.mdpi.com/1422-0067/19/7/1940/s1.

## Abbreviations

HBV	Hepatitis B virus
HBsAg	HBs antigen
HBeAg	HBe antigen
Peg-IFN	Pegylated interferon
NUCs	Nucleos(t)ide analogues
ETV	Entecavir
LMD	Lamivudine
ADV	Adefovir
miRNA	MicroRNA
FDR	False discovery rate
NTCP	Sodium taurocholate cotransporting polypeptide
NC	Negative control
Fib-4 index	Fibrosis-4 index
