# Supplementary material for: Serum miRNAs Predicting Sustained HBs Antigen Reduction 48 Weeks after Pegylated Interferon Therapy in HBe Antigen-Negative Patients
Source: Int J Mol Sci. 2018 Jul 2;19(7):1940. doi: 10.3390/ijms19071940 (PMC6073286; doi:10.3390/ijms19071940)
Supplement: Supplementary file 1 [file ijms-19-01940-s001.pdf]

Figure S1

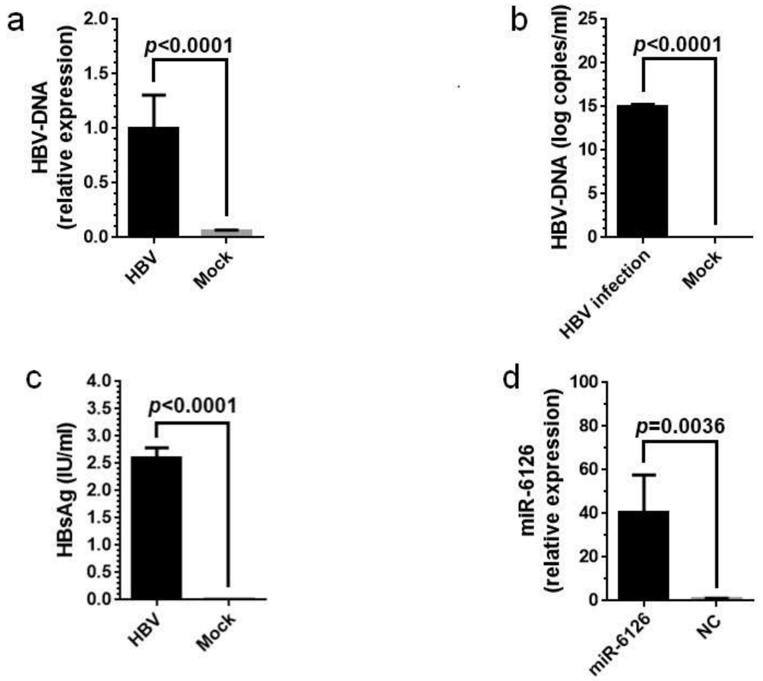

Figure S1. (A-C) Infection of Huh7/NTCP cells with HBV resulted in significantly larger quantities of intracellular HBV-DNA (A), and higher levels of HBV-DNA (B) and HBsAg (C) in culture medium supernatants than did mock infection ($p<0.05$). (D) Transfection of miR-6126 mimic resulted in significantly larger quantities of intracellular miR-6126 than did transfection of negative control (NC) ($p<0.05$). In the experiments (A-C), Huh7/NTCP cells were passaged on day 0; cells were infected with HBV on day 1; and cellular DNA and culture medium supernatants were harvested on day 4. Intracellular HBV-DNA quantities were normalized to those of GAPDH, an internal control. In the experiment (D), Huh7/NTCP cells were passaged on day 0; miR-6126 mimic or NC mimic was transfected into cells on day 1; total RNA was harvested on day 3. Intracellular quantities of miR-6126 were normalized to those of U6, an internal control for intracellular miRNAs. All the experiments (A-D) were performed three times with four replicates, then analyzed with Student's *t*-tests, and representative data are plotted with the mean and SD.
